# A review of Bruton’s tyrosine kinase inhibitors in multiple sclerosis

**DOI:** 10.1177/17562864241233041

**Published:** 2024-04-17

**Authors:** Laura Airas, Robert A. Bermel, Tanuja Chitnis, Hans-Peter Hartung, Jin Nakahara, Olaf Stuve, Mitzi J. Williams, Bernd C. Kieseier, Heinz Wiendl

**Affiliations:** Division of Clinical Neurosciences, University of Turku, Turku, Finland; Neurocenter, Turku University Hospital, Turku, Finland; Mellen Center for MS, Neurological Institute, Cleveland Clinic, Cleveland, OH, USA; Brigham Multiple Sclerosis Center, Harvard Medical School, Boston, MA, USA; Department of Neurology, Medical Faculty, Heinrich-Heine-University, Düsseldorf, Germany; Brain and Mind Center, University of Sydney, Sydney, NSW, Australia; Department of Neurology, Palacký University Olomouc, Olomouc, Czech Republic; Department of Neurology, Keio University School of Medicine, Tokyo, Japan; Department of Neurology, University of Texas Southwestern Medical Center, Dallas, TX, USA; Neurology Section, VA North Texas Health Care System, Dallas, TX, USA; Peter O’Donnell Brain Institute, University of Texas Southwestern Medical Center, Dallas, TX, USA; Joi Life Wellness MS Center, Atlanta, GA, USA; Department of Neurology, Medical Faculty, Heinrich-Heine-University, Düsseldorf, Germany; Novartis Pharma AG, Basel, Switzerland; Department of Neurology, University Hospital Muenster, Albert-Schweitzer-Campus 1, Building A 1, Muenster 48149, Germany

**Keywords:** BTK inhibitors, disease-modifying therapies, long-term administration, multiple sclerosis, safety, selectivity

## Abstract

Bruton’s tyrosine kinase (BTK) inhibitors are an emerging class of therapeutics in multiple sclerosis (MS). BTK is expressed in B-cells and myeloid cells, key progenitors of which include dendritic cells, microglia and macrophages, integral effectors of MS pathogenesis, along with mast cells, establishing the relevance of BTK inhibitors to diverse autoimmune conditions. First-generation BTK inhibitors are currently utilized in the treatment of B-cell malignancies and show efficacy in B-cell modulation. B-cell depleting therapies have shown success as disease-modifying treatments (DMTs) in MS, highlighting the potential of BTK inhibitors for this indication; however, first-generation BTK inhibitors exhibit a challenging safety profile that is unsuitable for chronic use, as required for MS DMTs. A second generation of highly selective BTK inhibitors has shown efficacy in modulating MS-relevant mechanisms of pathogenesis in preclinical as well as clinical studies. Six of these BTK inhibitors are undergoing clinical development for MS, three of which are also under investigation for chronic spontaneous urticaria (CSU), rheumatoid arthritis (RA) and systemic lupus erythematosus (SLE). Phase II trials of selected BTK inhibitors for MS showed reductions in new gadolinium-enhancing lesions on magnetic resonance imaging scans; however, the safety profile is yet to be ascertained in chronic use. Understanding of the safety profile is developing by combining safety insights from the ongoing phase II and III trials of second-generation BTK inhibitors for MS, CSU, RA and SLE. This narrative review investigates the potential of BTK inhibitors as an MS DMT, the improved selectivity of second-generation inhibitors, comparative safety insights established thus far through clinical development programmes and proposed implications in female reproductive health and in long-term administration.

## Plain language summary

A review of Bruton’s tyrosine kinase inhibitors in multiple sclerosis

**Why was this study done?** This study was done to find out about current knowledge on a type of drug, called Bruton’s tyrosine kinase inhibitors, or BTK inhibitors. There are currently six BTK inhibitors being studied as a possible new drug for treating multiple sclerosis (MS). Some of these six drugs are also being studied as a possible new drug for chronic spontaneous urticaria, rheumatoid arthritis and systemic lupus erythematosus. These are all autoimmune conditions, where the immune system mistakenly attacks parts of the body. Clinician scientists wanted to understand what is currently known about BTK inhibitors, how they work in the laboratory and how safe they could be for treating autoimmune conditions. This could help us understand more about BTK inhibitors in MS.**What did the scientists do?** The scientists assessed existing research on these six BTK inhibitors, through a process known as a literature review. These were results from ongoing clinical trials, and information collected through studying BTK inhibitors in laboratories. The researchers pieced together all these findings, to produce this paper that summarizes the results.**What did the scientists find?** The scientists found that most studies of BTK inhibitors for MS are still ongoing. So far, BTK inhibitors seem to show reasonable safety in most studies, but it is too early to know. The researchers also found out about how BTK inhibitors work in the lab, about what could happen if the drugs are taken for a long time and how they could impact female reproductive health.**What do these findings mean?** These findings will help other scientists learn more about BTK inhibitors in MS. Trials with BTK inhibitors for MS are still ongoing, but piecing together all the current findings gives a picture of what we know and what still needs to be done.

## Introduction

Bruton’s tyrosine kinase (BTK) is a non-receptor, cytoplasmic tyrosine kinase expressed by, and critical for, the development of B-cells and myeloid cells, located both peripherally and within the central nervous system (CNS).^1,2^ Inhibitors of BTK, such as ibrutinib, acalabrutinib and zanubrutinib,^3,4^ are approved for the treatment of B-cell malignancies, suggesting efficacy upon B-cell inhibition.^2,3^ In mature B-cells, BTK is a signal transducer downstream from toll-like receptor 4^5^ and B-cell receptor.^6^ B-cell activation is an integral pathogenic mechanism of chronic autoinflammatory conditions; thus, efficacy in B-cell inhibition could establish a relevance for BTK inhibitors in the management of autoinflammatory conditions.^1^ Furthermore, BTK is expressed in the myeloid cells, specifically the macrophages,^7^ dendritic cells,^8^ mast cells^9^ and microglia,^10^ acting downstream from Fcγ and Fcε receptors,^2,10^ establishing its relevance in diverse autoinflammatory mechanisms of pathogenesis. However, ibrutinib, acalabrutinib and zanubrutinib have exhibited significant off-target effects, including increased risk of haemorrhage, infection and atrial fibrillation.^4,11^ This generation of BTK inhibitors may, therefore, be unsuitable for long-term use, as required for chronic inflammatory conditions, owing to their challenging safety profiles. Therefore, development of the next generation of BTK inhibitors, with improved specificity and resultant tolerability is needed. Currently, second-generation BTK inhibitors are under investigation for multiple autoimmune conditions, including systemic lupus erythematosus (SLE), rheumatoid arthritis (RA), chronic spontaneous urticaria (CSU) and multiple sclerosis (MS).^1,12^ Incorporating learning from ongoing clinical trials, this review will evaluate the potential of second-generation BTK inhibitors, their improved selectivity and anticipated safety profile in the treatment of MS.

MS is a chronic, immune-mediated disease of the CNS, leading to demyelination and axonal damage.^13^ This translates into clinical disability for people living with MS.^13,14^ Second-generation BTK inhibitors are under clinical development for relapsing MS (RMS), relapsing-remitting MS (RRMS), primary progressive MS (PPMS) and one in secondary progressive MS (SPMS), summarized in Table 1. In MS pathogenesis, T-cells, B-cells and myeloid cells of the immune system play a key role in damaging the CNS and, as such, they have been of much interest as a therapeutic target in the development of disease-modifying treatments (DMTs).^13,15^ Immune-mediated mechanisms of inflammation are of much importance in MS progression; thus, targeting these mechanisms is integral in the development of prospective DMTs for multiple MS subtypes.^16^ The efficacy of recent B-cell depleting DMTs in MS management has further highlighted the role and importance of B-cells in the pathogenesis of this disease.^17^ An unmet need for new treatments remains, despite the availability of multiple DMTs for MS.^18^ Owing to the heterogeneity of MS, the use of specific DMTs may be limited among individuals, depending upon comorbidities, life situation, drug tolerance and toxicities^14,18^; thus, further treatments need to be developed. Specific DMT use may also include patient preferences owing to tolerability and administration methods. Second-generation BTK inhibitors may hold some potential, with prospective transience of treatment effects, potential for flexible dosing and oral administration differing from current B-cell depleting therapeutics.^1^

**Table 1.** Current clinical development and characteristics of BTK inhibitors for MS.

Trial information				BTK inhibitor characteristics		
BTK inhibitor	Lead developer	Current phase of development	Subtype of studied MS	BTK binding mechanism	Half-life (h)^a^	IC_50_ *in vitro* (nM)^b^
BIIB091	Biogen	II	RMS	Non-covalent	0.66	87
Evobrutinib	Merck KGaA	III	RRMS, SPMS with relapses	Covalent	2	47.7
Fenebrutinib	Roche	III	RMS, PPMS	Non-covalent	4.2–9.9	5.3
Orelabrutinib	Innocare	II	RRMS	Covalent	1.5–4	1.6
Remibrutinib	Novartis	III	RMS	Covalent	1–2	5.5
Tolebrutinib	Sanofi/Principia	III	RMS, SPMS, PPMS	Covalent	1.5–2	0.9

a Half-life data obtained from pharmacodynamic clinical trials, evobrutinib,^19^ fenebrutinib,^20^ remibrutinib^21^ and tolebrutinib.^22^

b IC_50_ data for evobrutinib, fenebrutinib, remibrutinib and tolebrutinib were obtained from comparative analyses research article;^1^ data for orelabrutinib^23^ were obtained from recent clinical trials; data for BIIB091 were obtained from *in vitro* studies.^24^

BTK, Bruton’s tyrosine kinase; MS, multiple sclerosis; PPMS, primary progressive multiple sclerosis; RMS, relapsing multiple sclerosis; RRMS, relapsing-remitting multiple sclerosis; SPMS, secondary progressive multiple sclerosis.

## Evidence identification

Literature for this review was identified through a provisional scoping search, followed by targeted literature searches through the Embase database *via* the Ovid platform for clinical trial information, publications and conference posters. This process followed best practice guidance in the development of narrative reviews.^25^ Literature searching focused on three themes using pre-defined eligibility criteria and identified evidence relating to BTK inhibition in autoimmune conditions, MS and BTK inhibitor safety profiles. Search terms included ‘Bruton’s tyrosine kinase inhibitor’ and ‘multiple sclerosis’, ‘autoimmune’ and associated drug safety terms. This was also conducted for the six BTK inhibitors in phase II or III trial for MS, using the search terms ‘Multiple Sclerosis’ and ‘BIIB091’, ‘evobrutinib’, ‘fenebrutinib’, ‘orelabrutinib’, ‘remibrutinib’ and ‘tolebrutinib’. The titles and abstracts of search results were screened by a single reviewer for relevance for these sections.

## BTK-relevant mechanisms of MS pathogenesis

**Figure 1.**
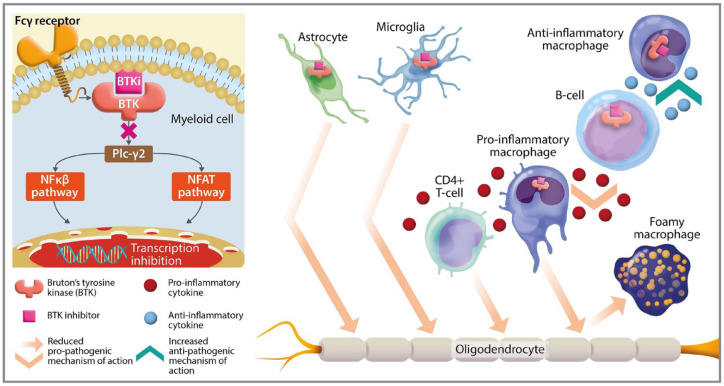
Simplified mechanisms of MS pathogenesis relevant to BTK inhibition. Proposed, simplified, MS-relevant BTK molecular mechanism of action following inhibition. In myeloid cells, BTK is activated through Fcγ receptor signalling and then signals through Plc-γ2 to activate the NFĸβ and NFAT pathway, leading to transcriptional activation. In B-cells, BTK is activated through B-cell receptor signalling; BTK inhibition may increase pro-inflammatory cytokine production. BTK is expressed in myeloid cells, with important roles in their function,^2,6^ including brain-specific microglia, and peripheral macrophages.^29^ In MS pathogenesis, B-cells can undergo antigen-triggered activation, a BTK-mediated pathway, to release pro-inflammatory cytokines including IFNƴ and TNFα.^2^ Release of pro-inflammatory cytokines can lead to the differentiation of pro-inflammatory CD4+ T-cells, implicated in demyelination.^30^ Pro-inflammatory cytokine release from B-cells also leads to macrophage polarization, promoting transformation to the pro-inflammatory M1 phenotype.^5^ These have been implicated in myelin phagocytosis, resulting in foam cells, which have been found in active neuronal lesions in MS.^31,32^ BTK is also expressed in the microglia, CNS-specific macrophages, leading to microglial activation.^33^ Activated microglia are also implicated in demyelination and are abundant in active neuronal MS lesions.^10,33^ Inhibition of BTK may have potential in the mediation of these mechanisms,^10^ thus it is of interest for novel MS therapeutics. BCR, B-cell receptor; BTK, Bruton’s tyrosine kinase; CNS, central nervous system; IFNƴ, interferon gamma; MS, multiple sclerosis; NFAT, nuclear factor of activated T cells; NFκβ, nuclear factor kappa-light-chain-enhancer of activated B cells; TNFα, tumour necrosis factor alpha.

Inhibitors of BTK have several proposed mechanisms of action, which may be relevant to the pathogenesis of MS,^2,26^ as summarized in Figure 1. Pre-clinical models have been utilized to investigate the role of BTK in MS. Treatment with the BTK inhibitor evobrutinib (Merck KGaA) dose-dependently inhibited maturation of B-cells, antigen-triggered activation and the release of pro-inflammatory cytokines in animal models of MS. This resulted in reduced disease severity in mice, highlighting the potential importance of BTK signalling pathways in MS pathogenesis. Furthermore, leptomeningeal inflammation, which was associated with poor clinical outcomes in MS, has been modelled in experimental autoimmune encephalomyelitis (EAE) mice.^27^ Mice treated with evobrutinib showed a significant improvement in meningeal inflammation, compared with vehicle, exhibiting a 30% reduction and a 5% increase, respectively.^27^ Remibrutinib (Novartis) treatment in EAE models strongly reduced B-cell-dependent inflammation and exhibited BTK occupancy in the brain and peripheral immune system.^28^

In *Xenopus* transgenic models, approximately 75% of immune cells expressing BTK were microglia, and the remainder astrocytes, suggesting a high bioavailability of BTK in the CNS of this model system.^15^ Microglia initiate demyelination and immune filtration in MS pathogenesis during the early phases of the condition, while in later stages they can promote both neurodegeneration and remyelination.^34^ Microglia BTK could be an important therapeutic target, if the pro-inflammatory function could be curtailed in early phases of the condition and the pro-remyelinating function promoted. In the *Xenopus* transgenic model, *in vivo* BTK inhibition resulted in a 1.7-fold improvement in remyelination, compared with untreated controls.^10^ Furthermore, *in vitro* and *in vivo* mouse models demonstrated that BTK expression is increased upon microglia activation and treatment with the BTK inhibitor evobrutinib reduces the expression of inflammatory surface markers, implicating a BTK inhibition effect upon microglia functionality.^10,28^ The role of BTK in systemic immune activation, as well as its potential impact on microglia suggests that BTK inhibition represents a promising treatment pathway for MS. This may be pertinent in MS subtypes, such as PPMS and SPMS, in which progression independent of relapse activity, partly driven by microglial activation, may be a key contributor.^35^

### Clinical insights of BTK mode of action in MS

There are six BTK inhibitors currently under development for the treatment of MS. These include: BIIB091 (Biogen, Cambridge, Massachusetts, United Stated), evobrutinib (Merck KGaA, Darmstadt, Germany), fenebrutinib (Roche, Basel, Switzerland), orelabrutinib (Innocare, Beijing, China), remibrutinib (Novartis, Basel, Switzerland) and tolebrutinib (Sanofi, Paris, France). A summary of different clinical trials, target population and BTK inhibitor characteristics are provided in Table 1.

Early phase clinical trials have revealed that BTK inhibitors can be detected in the cerebrospinal fluid (CSF) after oral administration. It still needs to be established to what extent CSF concentration reflects BTK tissue occupancy in the CNS and prediction of clinical efficacy. Tolebrutinib exhibited dose-dependent peripheral BTK occupancy in the blood and entered the CSF in phase I trials.^22^ In a phase IIb trial of tolebrutinib, a dose-dependent reduction in new gadolinium-enhancing lesions in the brain could be demonstrated,^36^ indicating the potential efficacy of BTK inhibition in modulating CNS inflammatory activity.^37^

A phase II trial of evobrutinib, which was placebo- and dimethyl fumarate- controlled, illustrated that participants receiving 75 mg dosage of evobrutinib had significantly fewer gadolinium-enhancing lesions upon MRI 12–24 weeks after treatment initiation compared with the placebo group.^38^ Similarly, 12 week interim analyses of an orelabrutinib phase II trial demonstrated reductions in gadolinium-enhancing lesions in participants receiving orelabrutinib compared with placebo.^39^ Future clinical studies will seek to demonstrate additional clinical benefits in MS. Great therapeutic focus will rest upon the modulation of microglia and myeloid cells by BTK inhibitors. This is particularly pertinent when BTK expression is considered, because *in vivo* models have suggested that, proportionally, there is higher expression and potentially bioavailability of BTK in the microglia compared with astrocytes.^10^ Targeting of microglia alongside B cells could be beneficial as effectors of MS pathogenesis.^34^

## Second-generation BTK inhibitor binding mechanisms

For long-term use in chronic autoinflammatory conditions, such as MS, the binding mechanisms of BTK inhibitors need to be considered. First-generation BTK inhibitors currently approved for use in oncology or second-generation BTK inhibitors under investigation in MS, with the exception of BIIB091 and fenebrutinib, are covalent inhibitors.^4^ Covalent inhibitors of BTK target the SH3 pocket of BTK, irreversibly binding to cysteine 481 (Cys481) and, therefore, blocking kinase activation.^40^ First-generation BTK inhibitor ibrutinib is a covalent inhibitor, utilized in the treatment of B-cell malignancies, mantle cell lymphoma (MCL) and chronic lymphoid leukaemia (CLL).^3^ In a subset of people with CLL or MCL, resistance to ibrutinib has been noted, reducing the potency of BTK inhibition,^41,42^ highlighting a potential challenge in long-term BTK inhibitor use. Notably, resistance to therapeutics is relatively common in oncology, owing to the enhanced proliferative rate of malignant cells, selection pressure caused by therapeutics and resultant selective advantage for resistance mutations.^3,6,43^ This may not be the case in the use of BTK inhibitors for non-malignant autoimmune conditions such as MS. However, resistance should be considered and monitored in the development of covalent BTK inhibitors for long-term use.

Non-covalent inhibitors reversibly bind to different BTK-specific pockets.^41,44^ As an example of a non-covalent BTK inhibitor, BIIB091 (Biogen) is an orthosteric, reversible adenosine triphosphate competitor, which sequesters tyrosine 551, an important BTK phosphorylation site, leading to an inactive conformation.^44^ A phase II trial examining BIIB091 in RMS is planned and yet to recruit, while fenebrutinib is undergoing a phase III clinical trial for people with RMS or PPMS,^45^ ClinicalTrials.gov identifiers: NCT05798520, NCT04586010, NCT04544449. The transient binding mechanics displayed by non-covalent BTK inhibitors may provide advantages in terms of long-term administration^41,44^; however, the distinction between covalent and non-covalent BTK inhibitors is not yet fully understood, nor have these differences been established in the context of MS.

## Second-generation BTK inhibitor selectivity

Overall, the second-generation BTK inhibitors under investigation for the treatment of MS have low half maximal inhibitory concentration (IC_50_) values for BTK binding, as described in Table 1, suggesting high selectivity. A recent *in vitro* comparative study, investigating B-cell inhibition further implicated this previously observed high selectivity, with IC_50_ values after 1 h of 18 nM for remibrutinib, 320 nM for evobrutinib, 74 nM for tolebrutinib, 185 nM for orelabrutinib and 15 nM for fenebrutinib.^46^ Subsequent kinome screening at 1 µM illustrated a ranking from the least to most off-target kinase binding. The least off-target binding was shown by remibrutinib, followed by fenebrutinib, evobrutinib, orelabrutinib and tolebrutinib.^46^ These data suggest varied but promising selectivity, which may have downstream implications in the off-target side effects and safety profile of these BTK inhibitors. BIIB091 was not included in these analyses, but has an *in vitro* IC_50_ value of 87 nM in stimulated B cells and 106 nM in myeloid cells.^24^

The IC_50_ for many of the BTK inhibitors has been reported for closely related kinases of the tyrosine-protein kinase (TEC) family, of which BTK is a member, including TEC, interleukin-2-inducible T-cell kinase (ITK), cytoplasmic tyrosine-protein kinase (BMX) and tyrosine-protein kinase (TXK).^1^ Tolebrutinib has a comparable IC_50_ for TEC, BMX and TXK, but an IC_50_ more than 350-fold higher for ITK, when compared with BTK and TEC.^1^ Conversely, fenebrutinib shows an improved cross selectivity profile, exhibiting IC_50_ values above 1000 nM for TEC, ITK and TXK.^1^ Similarly and despite its comparably high IC_50_ value for BTK, evobrutinib has IC_50_ values greater than 1000 nM for ITK and 100 nM for TKX, respectively.^1^ Data on cross selectivity for these kinases are not available for remibrutinib and orelabrutinib. However, a screen for BTK inhibition of 218 off-target kinases demonstrated that in terms of IC_50_ values, fenebrutinib is over 130-fold more selective than evobrutinib and tolebrutinib. Furthermore, fenebrutinib dissociation from BTK has been shown to be slow, with a residence time of 18.3 h, implicating high stability.^47^ The clinical relevance of differences in selectivity and stability among inhibitors may also translate in ongoing clinical trials, but this is yet to be fully investigated.

In comparison, the first-generation BTK inhibitor ibrutinib shows an >80% occupancy of all of these closely related TEC kinases, along with important receptor and intracellular kinases such as epidermal growth factor receptor (EGFR), human epidermal growth factor receptor 2 (HER2), human epidermal growth factor receptor 4 (HER4) and Janus kinase 3.^4^ Off-target effects of HER2 and HER4 inhibition include atrial fibrillation,^4^ which has been reported in ibrutinib treatment.^12^ Commonly reported adverse events (AEs) in ibrutinib treatment are rash and diarrhoea, previously thought to be through inhibition of EGFR; however, recent data have illustrated this to be inconclusive.^4^

Ibrutinib is currently the only BTK inhibitor approved by the US Food and Drug Administration utilized for autoimmunity in steroid-resistant chronic graft *versus* host disease (cGvHD).^48^ A phase II clinical trial for cGvHD demonstrated high efficacy, with 67% of participants showing an objective response rate.^12^ However, low-grade infection AEs were common, experienced by 69% of participants,^12^ while two participants in an evaluable population of 42 died as a result of AEs, pneumonia and bronchopulmonary aspergillosis.^12^ These infectious complications may be due to immunosuppression during ibrutinib treatment. Furthermore, treatment with ibrutinib has been illustrated to impair the immune response to influenza, hepatitis C and zoster vaccines in people with CLL.^49,50^ The effect of treatment upon immune response to both infections and vaccines may be a key consideration in the development of second-generation BTK inhibitors for MS.^51^

Encouragingly, second-generation BTK inhibitors including evobrutinib, fenebrutinib and remibrutinib under trial for MS broadly show <50% occupancy of TEC kinases.^4^ These data suggest that recently developed BTK inhibitors demonstrate a promising selectivity profile; however, this is yet to be fully established. Off-target effects, which could be attributed to binding to other members of the TEC kinase family, may manifest during clinical trials of BTK inhibitors. The TEC kinases have similar roles to BTK, with high expression in myeloid cells,^1^ implicating the possibility of immunity-related AEs. However, after the challenging safety profiles of first-generation BTK inhibitors, there is likely to be a cautious approach to safety in the use of second-generation BTK inhibitors for MS, along with a range of autoimmune conditions.

## Concentration of BTK inhibitors in the CNS and adjacent CSF

Much of the pharmacokinetic and pharmacodynamic data available for second-generation BTK inhibitors are based on blood absorption and peripheral target occupancy, with little data available regarding the concentration of BTK inhibitors in the CSF. Furthermore, pharmacokinetic studies undertaken in phase I do not reflect dosages given in phase II and III trials. A phase I trial of tolebrutinib demonstrated that 2 h after a first dose of 120 mg, there was a mean CSF concentration of 1.87 ng/mL across four healthy participants.^22^ However, this time point is in conflict with the half-life of tolebrutinib of between 90 and 120 min; therefore, the study team highlighted that the actual CSF concentration may be higher; thus, further research on this with more sensitive time points and lower dosages utilized in clinical development is needed.^22,52^ Subsequent studies in healthy volunteers suggested that tolebrutinib remains present at bioactive levels in the CSF 4 h after dosing, when given at dosages of 60 or 120 mg.^53^ Similarly, CSF concentration has been measured for evobrutinib, reaching a mean concentration of 3.21 ng/mL 2 h post dose, across nine participants with RRMS.^54^ A previous study also highlighted that tolebrutinib administration resulted in alterations in CSF protein levels in people with MS, compared with untreated people with MS.^55^ This further suggests tolebrutinib presence in the CSF in people with MS. The presence of BTK inhibitors in the CSF implicates potential adjacent CNS relevant mechanisms of action to be further elucidated.

The suitability of second-generation BTK inhibitors for the management of MS will be illuminated through further dissection of the CSF exposure through determination of drug concentration. The extent to which CSF concentration reflects tissue occupancy in the CNS and subsequently predicts clinical efficacy still needs to be established.

## Safety summary of second-generation BTK inhibitors in MS

Transferable insights may be obtained regarding safety, efficacy and mechanisms of action, which could be relevant to MS, through understanding the development and trial results of BTK inhibitors in other autoimmune conditions. Phase II and III clinical trials investigating the efficacy of BTK inhibitors for diverse autoimmune conditions are currently underway. The key safety and efficacy findings of these trials can be found in Table 2.

**Table 2.** Summary of BTK inhibitors under clinical trial for autoimmune conditions.

Trial information				Summary of key findings	
Autoimmune condition	BTK inhibitor	Lead developer	Current phase of development	Safety	Efficacy
Chronic spontaneous urticaria	Remibrutinib	Novartis	III	• No dose-limiting toxicities observed in dosages up to 600 mg per day in phase I • High tolerability exhibited • Pharmacodynamic data support high selectivity	• Dose-dependent improvements in symptoms following 12 weeks of treatment in phase IIb
	Fenebrutinib	Roche	II-discontinued	• Well tolerated, with small proportion of mild AEs • Most common AEs were urticaria, nasopharyngitis and headache	• Reduction of autoantibodies, suggesting BTK target engagement • Improvement of disease activity by week 8 at selected dosages • Findings from interim analyses of efficacy data lead to trial discontinuation
Rheumatoid arthritis	Evobrutinib	Merck KGaA	IIb	• High tolerability exhibited, with comparable treatment-emergent AEs among groups	• Phase IIb trials failed to reach the primary endpoint of ACR20, a measure indicating a >20% improvement of participants’ RA symptoms
	Fenebrutinib	Roche	II	• Clinical trial and OLE analyses for fenebrutinib in multiple autoimmune conditions suggests high tolerability • Phase II studies highlighted favourable safety profile	• Dose-dependent improvements in ACR50, a measure indicating a >50% improvement of participants’ RA symptoms • Trials in population, unresponsive to TNF therapeutics or methotrexate, taking methotrexate in combination with fenebrutinib showed improvements in ACR50
Systemic lupus erythematosus	Evobrutinib	Merck KGaA	II-	• Well tolerated and favourable safety profile shown	• Failed to meet primary efficacy endpoints
	Fenebrutinib	Roche	II-discontinued	• Was well tolerated	• Failed to meet primary efficacy endpoints • Reduction of total IgG and IgM antibody levels by week 48, suggesting target engagement

Summary of key safety and efficacy findings in clinical trials of BTK inhibitors for autoimmune conditions. These BTK inhibitors are also under trial for MS. Data obtained to populate Table 2 were obtained from clinical trial findings, published primary research articles and conference proceedings. Further details can be found in the references as follows. Chronic spontaneous urticaria: remibrutinib,^21,56^ fenebrutinib, ClinicalTrials.gov identifier: NCT03693625, NCT03137069.^57^ RA: evobrutinib,^58^ fenebrutinib.^59,60,61,62^ Systemic lupus erythematosus: evobrutinib,^1,19,63^ fenebrutinib, ClinicalTrials.gov identifier: NCT03137069.^1,64^

ACR20, American College of Rheumatology response criteria 20; ACR50, American College of Rheumatology response criteria 50; AE, adverse event; BTK, Bruton’s tyrosine kinase; IgG, immunoglobulin G; IgM, immunoglobulin M; OLE, open-label extension; RA, rheumatoid arthritis; TNF, tumour necrosis factor.

As described in Table 1, there are six BTK inhibitors under investigation for MS. These are currently in phase II or III trials and have a targeted focus in treating people with RMS or RRMS, ClinicalTrials.gov identifiers: NCT05798520, NCT04586010, NCT04544449, NCT04338022, NCT04338061, NCT04410978, NCT04410991, NCT04411641, NCT04458051, NCT04711148, NCT04586023 and NCT05147220. Two phase III trials are underway for BTK inhibitors in PPMS and one phase III trial in non-relapsing SPMS, ClinicalTrials.gov identifiers: NCT04544449, NCT04458051 and NCT04411641. Current clinical trial developments and efficacy endpoints are summarized in Table 3, owing to the differences in MS population, endpoints and current stages between trials. The safety profiles of each BTK inhibitor will be summarized and contrasted. It is important to caveat that in terms of trial design, trial stage, patient populations, pharmacokinetic and pharmacodynamic properties, there are substantial differences between each BTK inhibitor; thus, they cannot be directly compared, or findings extrapolated in terms of safety.

**Table 3.** Summary of safety and efficacy of BTK inhibitors in MS clinical trials.

Trial information				Key findings	
BTK inhibitor	Lead developer	Current phase of development	Subtype of MS	Safety	Efficacy
BIIB091	Biogen	II	RMS	• Phase II trials are not yet recruiting	• Phase II trials are not yet recruiting
Evobrutinib	Merck KGaA	III	RRMS, SPMS with relapses	• Phase II trials demonstrated similar AE incidence across placebo and evobrutinib doses; however, grade 3–4 AEs were highest in the 75 mg daily and 75 mg twice daily groups. This was comparable with the dimethyl fumarate group	• Phase II trials have demonstrated a dose-dependent reduction in the expansion of slowly expanding lesions in participants with RRMS • High evobrutinib dosages compared with low dosages lead to significant reductions in slowly expanding lesions in participants with RRMS >8.5 years post-diagnosis and baseline EDSS score of >3.5
Fenebrutinib	Roche	III	RMS, PPMS	• OLE studies have suggested reduced frequency of AEs, the most common being nasopharyngitis, nausea and headache, following continuation of treatment • High tolerability shown in phase II trials, with no increased incidence of infection compared with placebo	• Efficacy endpoints for phase III will include sensitive measurement of MS symptomatic progression
Orelabrutinib	Innocare	II	RRMS	• Repurposed oncology drug for B-cell malignancies. Safety data from oncology suggests high incidence of AEs, but well tolerated	• Phase II trials are ongoing • Interim analyses at 12 weeks have illustrated a dose-dependent reduction of new brain lesions
Remibrutinib	Novartis	III	RMS	• Favourable safety profile and high tolerability shown in CSU	• Phase II findings currently unavailable • Recruiting participants with RMS for phase III trials
Tolebrutinib	Sanofi/Principia	III	RMS, SPMS, PPMS	• No serious AEs in phase I trials. Mild AEs occurring in >10% of participants were headache and diarrhoea • Phase IIb: there were no discontinuations relating to treatment	• Phase IIb demonstrated dose-dependent reductions of gadolinium-enhancing lesions after 12 weeks of treatment in participants with RMS • Phase III trials underway for participants with RMS, SPMS and PPMS

Summary of key safety and efficacy findings in clinical trials of BTK inhibitors for the treatment of MS. Data obtained to populate Table 3 have been obtained from clinical trial findings, published primary research articles and conference proceedings. Further details can be found in the references as follows: BIIB091, evobrutinib,^65,66^ fenebrutinib,^59,67^ orelabrutinib,^68,39,69^ remibrutinib,^56,70^ tolebrutinib.^22,36^ ClinicalTrials.gov identifiers: NCT05798520, NCT04338022, NCT04338061, NCT04032158, NCT04586023, NCT04711148, NCT05147220, NCT04410978, NCT04410991, NCT04411641 and NCT04458051.

AE, adverse event; BTK, Bruton’s tyrosine kinase, CSU, chronic spontaneous urticaria; EDSS, Expanded Disability Status Scale; MS, multiple sclerosis; OLE, open-label extension; PPMS, primary progressive multiple sclerosis; RMS, relapsing multiple sclerosis; RRMS, relapsing-remitting multiple sclerosis SPMS, secondary progressive multiple sclerosis.

Tolebrutinib showed favourable safety exposure and pharmacodynamics in a phase I trial.^22^ For all participants, the drug was well tolerated with no serious AEs, while the pharmacokinetic profile exhibited rapid absorption and favourable CSF concentrations.^22^ Following on from this, a phase IIb trial of tolebrutinib treating RMS further highlighted the favourable safety profile, with no AE-related discontinuations reported in 130 participants.^36^ Safety signals in a phase III trial of reversible drug-induced liver injury in June 2022 placed the programme on partial hold and paused recruitment in trials across MS subtypes,^52^ including RMS, SPMS and PPMS, but trials have since resumed,^71^ ClinicalTrials.gov identifiers: NCT04410978, NCT04410991, NCT04411641 and NCT04458051. The majority of drug-induced liver injuries occurred in individuals with concurrent, predisposing medical conditions and resolved following treatment discontinuation.^52^ The safety challenges encountered in the phase III trial could potentially account for the reduced selectivity profile of tolebrutinib when compared with other second-generation BTK inhibitors in development.^46^ RMS is also the planned focus of a phase II trial of BIIB091, which is not yet recruiting ahead of initiation, ClinicalTrials.gov identifier: NCT05798520. Phase I safety data for BIIB091 are not yet publicly available, ClinicalTrials.gov identifier: NCT03943056. Similarly, orelabrutinib is a BTK inhibitor for B-cell malignancies and is currently under phase II trial for the treatment of RRMS only,^68^ ClinicalTrials.gov identifier: NCT04711148. A retrospective study highlighted that it is well tolerated in the treatment of CNS lymphoma.^69^

Evobrutinib is undergoing phase III trial for the treatment of RRMS or SPMS with relapses and has shown promising safety profiles in earlier phases, ClinicalTrials.gov identifiers: NCT04338022, NCT04338061 and NCT04032158. The first in-human phase I study of evobrutinib in terms of safety, tolerability and pharmacokinetics was undertaken with the view to develop it as a prospective therapeutic for SLE, RA and RRMS, demonstrating a favourable safety profile.^19^ A recent analysis of evobrutinib AEs illustrated that this BTK inhibitor is well tolerated in people with MS, utilizing data from phase II trials of evobrutinib for MS, RA and SLE from more than 1000 participants.^65^ The proportion of participants across all autoimmune conditions experiencing AEs was comparable between evobrutinib and placebo groups, with an incidence of 66.2% and 62.4%, respectively.^65^

A remibrutinib phase III clinical trial is also recruiting for people with RMS, ClinicalTrials.gov identifier: NCT05147220. Remibrutinib is a covalent BTK inhibitor, which has shown high *in vitro* target occupancy, suggesting high selectivity and potency.^70^ Like alternative BTK inhibitors, remibrutinib has shown a favourable safety profile across dosages in CSU.^56^ In 267 participants with CSU, no dose-dependent pattern in AEs was exhibited and most AEs were mild to moderate in severity.^72^ Remibrutinib has shown high efficacy in the treatment of CSU in a phase IIb study.^56^ Daily dosages of up to 600 mg were investigated in phase I, with no dose-limiting toxicities exhibited and high tolerability demonstrated.^21^ With dosages equal to or higher than 30 mg, blood BTK occupancy was greater than 95% for at least 24 h, further suggesting high selectivity and potency.^21^ This is supportive of the favourable safety profile exhibited and phase III trial of remibrutinib for CSU started in late 2021.^56^

A phase III study of fenebrutinib is currently recruiting people with RMS and PPMS, ClinicalTrials.gov identifier: NCT04586010, NCT04544449. High dosages of fenebrutinib were well tolerated, with no increase in infection rate, while AEs became less frequent following consistent administration in open-label extension (OLE) studies.^59^ In part, it could be speculated that this favourable safety profile, even at high dosages, could be due to the high selectivity of fenebrutinib demonstrated *in vitro*.^1^ These data supported the continuation of fenebrutinib trials in MS, enabling the initiation of phase III trials.^59,67^ Phase II trials and OLEs of fenebrutinib demonstrated a promising safety profile in a range of autoimmune conditions, including RA, SLE and CSU.^59^ Like remibrutinib, fenebrutinib has undergone a phase II trial for the treatment of CSU, ClinicalTrials.gov identifier: NCT03693625. However, this trial was discontinued in 2020, following an interim efficacy analysis, ClinicalTrials.gov identifier: NCT03137069. Despite this, fenebrutinib for the treatment of CSU exhibited high tolerability, with a small proportion of mild AEs, the most common being urticaria, nasopharyngitis and headache,^57^ ClinicalTrials.gov identifier: NCT03693625 and NCT03137069. The potential discrepancy between these two second-generation BTK inhibitors for CSU treatment in terms of efficacy suggests that there could be differences among inhibitors in their suitability for treating specific autoimmune conditions.

Although BTK inhibitors are receiving substantial attention as novel DMTs for MS, the results of ongoing phase III trials will be pivotal in evaluating whether the BTK inhibitors described demonstrate the desired benefit–risk profiles. Phase I and II trials have been promising; however, further conclusions cannot be drawn until the completion of phase III trials, which will enable a more thorough dissection of BTK inhibitor safety and efficacy.

### Potential implications of BTK inhibitor administration in female reproductive health

Worldwide, it is estimated that between 66% and 78% of people with MS are female, the average age of MS diagnosis across sexes is 32 years.^73^ Therefore, in the development of novel DMTs for MS, it is important to consider female needs in relation to reproductive health and choices.

A recent study in a healthy female study population investigating remibrutinib suggested that when administered at a dose of 100 mg twice daily, it does not substantially interfere with exposure of the combined oral contraceptive pill (COCP), containing 30 µg ethinylestradiol/150 µg levonorgestrel.^74^ There was no change in ethinylestradiol exposure, but a slight increase in levonorgestrel exposure.^74^ Furthermore, the incidence of AEs was higher when the COCP was administered alone (26.7%), than when both the COCP and remibrutinib were taken (22.2%),^74^ suggesting second-generation BTK inhibitor administration may not have pharmacodynamic or safety implications in COCP use. However, extrapolation of these data to a population with MS, across individual BTK inhibitors, hormonal contraceptive methods or COCP types should not be undertaken; thus, further research is needed.

First-generation BTK inhibitors such as ibrutinib are not recommended for use during pregnancy, with highly effective contraception required during administration, while second-generation BTK inhibitors have similar guidelines, ClinicalTrials.gov identifier: NCT04338022, NCT04338061, NCT04032158, NCT04410978, NCT04410991, NCT04411641, NCT04458051, NCT04711148, NCT04586023 and NCT05147220. To date, there have been no peer-reviewed articles detailing instances of pregnancy during BTK inhibitor clinical development and the effects of the drugs on a developing fetus are not known. As described in Table 1, BTK inhibitors have a short half-life, ranging from 1 to 9.9 h.^19–21^ This may make treatment with BTK inhibitors a flexible option for MS management, until close to stopping contraception for pregnancy planning. Post-partum and if breastfeeding is chosen, the concentration of MS DMTs in breastmilk and implications in infant exposure need to be considered.^75^ It is currently unknown whether second-generation BTK inhibitors are present in breastmilk,^75^ with participants who are pregnant or breastfeeding excluded from clinical trials. Further insights from prospective future OLE studies and real-world evidence may enable further elucidation of the implications of BTK inhibitors in the reproductive choices of people with MS.

### Potential implications of long-term BTK inhibitor administration

Despite the promising safety profiles of second-generation BTK inhibitors observed to date in MS trials, there are several adverse reactions that have previously been associated with first-generation BTK inhibitor administration.^4^ While not affecting all people taking BTK inhibitors, several adverse reactions are of concern when long-term administration is considered. Most notably, these include atrial fibrillation, dermatological toxicities, spontaneous bleeding and invasive fungal infection.^4^ Infectious complications could be due to potential immunosuppression, as previously discussed.^12,48–50^ Currently, it is unclear whether these effects are BTK inhibitor class-related and most prevalent in early therapeutics used short-term in the treatment of B-cell malignancies. Alternatively, non-infection-related reactions could be due to off-target effects.^76^ The BTK inhibitors currently under development have been purported to have a higher selectivity than those of the previous generation, as detailed throughout this review. Owing to the early development stage of second-generation BTK inhibitors for the treatment of MS, more clinical trial data are required to fully elucidate the safety profile in this patient population. Importantly, OLE studies and long-term administration will further enable the assessment of long-term impacts of BTK inhibitor administration and the safety profile of these treatments.

The effects of mid- to long-term use of BTK inhibitors in the treatment of MS are currently unknown. In humans, loss of function mutations and deletions of the *BTK* gene are responsible for the rare genetic condition X-linked agammaglobulinaemia, characterized by primary immunodeficiency, due to reduced circulation of B-cells and hypogammaglobulinaemia.^77^ The role of BTK in the maintenance of immunity should be considered in the development of inhibitors for long-term use, along with potential implications for immunosuppression from long-term administration.

## Conclusion

So far, the benefit–risk profile of second-generation BTK inhibitors in the treatment of MS is encouraging. BTK inhibitors can enter the CSF, thus highlighting the potential for access to cells of the adaptive and innate immune system, both in the CNS and periphery concurrently. Furthermore, preclinical models have suggested modulation of immune cells in demyelination and myelin repair following BTK inhibition, but this is yet to be understood in humans and may be illuminated by future clinical studies. The results of ongoing phase II and III trials will help to further elucidate the clinical efficacy and immune responses of BTK inhibition, along with understanding BTK inhibitor’s safety profile under chronic use, which will be observed more fully during OLE studies. It is yet to be established where in the therapeutic pathway BTK inhibitors would occupy, but this will be informed through ongoing clinical development and subsequent real-world evidence studies. The introduction of BTK inhibition was perceived as a major breakthrough in oncology; it is hoped that these drugs will bring a similar incremental value to the therapeutic armamentarium in fighting MS.

## Declarations

**Ethics approval and consent to participate:** Not applicable.

**Consent for publication:** Not applicable.

**Competing interests:** LA has obtained institutional research support from Aatos Erkko Foundation, Finnish Academy, Merck, Novartis, Sanofi Genzyme and US National MS Society, and compensation for lectures and advising from Janssen, Merck, Novartis, Roche, Sanofi Genzyme and Viracta is. TC has received compensation for consulting from Banner Life Sciences, Biogen, Bristol Myers Squibb, Novartis Pharmaceuticals, Roche Genentech and Sanofi Genzyme. She has received research support from the National Institutes of Health, National MS Society, US Department of Defense, Sumaira Foundation, Brainstorm Cell Therapeutics, Bristol Myers Squibb, EMD Serono, I-Mab Biopharma, Mallinckrodt ARD, Novartis Pharmaceuticals, Octave Bioscience, Roche Genentech, Sanofi Genzyme and Tiziana Life Sciences. JN received honoraria from Abbvie, Alexion, Amgen, Asahi-Kasei, Biogen, Bristol Myers, Chugai, CSL-Behring, Daiichi-Sankyo, Eisai, Kyorin, Mitsubishi-Tanabe, Novartis, Otsuka, Roche, Takeda and Teijin; received research supports from Biogen, Boehringer Ingelheim, Chugai, Daiichi-Sankyo, EA Pharma, Eisai, JB, MEXT, Mitsubishi-Tanabe, Otsuka, Shionogi, Sumitomo Pharma, Takeda and Teijin; serves as a paid scientific advisor to Alexion, Biogen, Chugai, Horizon, Roche and Novartis; serves as a local principal investigator for Novartis (remibrutinib) and Sanofi (tolebrutinib) and serves as a member of scientific steering committee for Novartis (remibrutinib). OS is an Associate Editor at *Therapeutic Advances in Neurological Disorders*; therefore, the peer review process was managed by alternative members of the Board and the submitting Editor was not involved in the decision-making process. In addition, OS has served on data monitoring committees for Genentech-Roche, Pfizer, Novartis and TG Therapeutics without monetary compensation, advised EMD Serono and VYNE, receives grant support from EMD Serono, is a 2021 recipient of a Grant for Multiple Sclerosis Innovation (GMSI), Merck KGaA, is funded by a Merit Review grant (federal award document number (FAIN) BX005664-01) from the United States (U.S.) Department of Veterans Affairs, Biomedical Laboratory Research and Development, and by RFA-2203-39314 (PI) and RFA-2203-39305 (co-PI) grants from the National Multiple Sclerosis Society (NMSS). HW has received honoraria for acting as a member of Scientific Advisory Boards from Abbvie, Alexion, Argenx, Bristol Myers Squibb/Celgene, Janssen, Merck, Novartis. He receives speaker honoraria and travel support from Alexion, Biogen, Bristol Myers Squibb, F. Hoffmann-La Roche Ltd, Genzyme, Merck, Neurodiem, Novartis, Roche Pharma AG, TEVA, WebMD Global. He is acting as a paid consultant for Abbvie, Actelion, Argenx, Biogen, Bristol Myers Squibb, EMD Serono, Fondazione Cariplo, Gossamer Bio, Idorsia, Immunic, Immunovant, Janssen, Lundbeck, Merck, NexGen, Novartis, PSI CRO, Roche, Sanofi, Swiss Multiple Sclerosis Society, UCB, Worldwide Clinical Trials. His research is funded by the German Ministry for Education and Research (BMBF), Deutsche Forschungsgesellschaft (DFG), Deutsche Myasthenie Gesellschaft e.V., Alexion, Amicus Therapeutics Inc., Argenx, Biogen, CSL Behring, F. Hoffmann-La Roche, Genzyme, Merck KGaA, Novartis Pharma, Roche Pharma, UCB Biopharma.

**Availability of data and materials:** Not applicable.
